# Utilizing proficiency testing survey data to create advanced educational content: the virtual crossmatch challenge model

**DOI:** 10.3389/fgene.2023.1256498

**Published:** 2023-09-21

**Authors:** Reut Hod-Dvorai, Mary Carmelle Philogene, Olga Timofeeva, Idoia Gimferrer, Heather Dunckley, Anna Greenshields, Peter Jindra

**Affiliations:** ^1^ Department of Pathology, SUNY Upstate Medical University, Syracuse, NY, United States; ^2^ Department of Surgery, Virginia Commonwealth University Medical Center, Richmond, VA, United States; ^3^ Department of Pathology, MedStar Georgetown University Hospital, Georgetown University Medical Center, Washington, DC, United States; ^4^ Department of Immunogenetics/HLA, BloodworksNW, Seattle, WA, United States; ^5^ New Zealand Transplantation and Immunogenetics Laboratory, New Zealand Blood Service, Auckland, New Zealand; ^6^ Department of Pathology, Dalhousie University, Halifax, NS, Canada; ^7^ Immune Evaluation Laboratory, Department of Surgery, Baylor College of Medicine, Houston, TX, United States

**Keywords:** proficiency testing, virtual crossmatch, HLA, transplant, HLA antibodies

## Abstract

Proficiency testing (PT) surveys include data from laboratories across the world and are ideal for creating advanced educational content, beyond just consensus grading. Educational challenges provide a unique opportunity to probe common laboratory practices and risk assessment, especially in cases where there is no “analyte” tested. Human leukocyte antigen (HLA) compatibility evaluation between donor and recipient pairs has been traditionally assessed using T-cell and B-cell physical crossmatches. However, advancements in our ability to identify and characterize HLA antibodies using solid phase assays, in combination with changing deceased donor allocation schemes and improved HLA typing, have shifted the paradigm from performing physical crossmatches to the use of the virtual crossmatch (VXM). VXM is a compatibility assessment relying on the interpretation of pre-transplant HLA laboratory data and as such, it is not an “analyte”. However, VXM results are used in clinical decision-making. The VXM assessment depends on patient characteristics as well as laboratory and transplant center practices but must ensure safe transplantation outcomes while maintaining equity in access to transplantation. In this manuscript, we describe the American Society for Histocompatibility and Immunogenetics (ASHI) PT Educational VXM Challenge, as a model for creating educational content using PT survey data. We discuss the different components of the VXM Challenge and highlight major findings and learning points acquired from ASHI VXM Challenges performed between 2018–2022, such as the lack of correlation between the VXM and the physical crossmatch in the presence of low level donor-specific antibodies (DSA), or when the DSA were aimed against donor alleles that are not present on the antibody panel, and in the presence of an antibody to a shared eplet. Finally, we show that the VXM Educational Challenge serves as a valuable tool to highlight the strengths and pitfalls of the VXM assessment and reveals differences in testing and result interpretation among participating HLA laboratories.

## Introduction

Histocompatibility laboratories around the world perform high complexity testing and must adhere to regulatory requirements by participating in proficiency testing (PT) for each analyte reported clinically. A preference is given for participation in graded external PT programs, however, when this requirement cannot be met, a laboratory can opt to participate in an ungraded PT program or use alternate mechanisms described in the American Society for Histocompatibility and Immunogenetics (ASHI) standards to validate test performance. PT programs provide blinded samples, collect test results, analyze the data, grade participants based on consensus and produce a summary of the results. PT survey results include data from laboratories across the world and are ideal for creating advanced educational content, beyond just consensus grading. This may be achieved by designing educational challenges which provide a unique opportunity to probe common laboratory practices and assessment of risk, especially in cases where there is no “analyte” tested.

Human leukocyte antigen (HLA) compatibility between donor and recipient pairs has been traditionally assessed using T-cell and B-cell physical crossmatches. T-cell and B-cell physical crossmatches are analytes that can be assessed by PT surveys and are often combined with HLA antibody identification testing. PT surveys for detection of anti-HLA antibody and physical crossmatching generally include 2 cell samples that are crossmatched against 4-5 serum samples. Of 156 laboratories that participated in the 2022 ASHI PT antibody and crossmatching (AC) survey, 68% are USA laboratories and the rest are international laboratories. Detailed demographics of the 2022 AC Survey participants can be found in Table 1. Among those participants, the most commonly reported physical crossmatch (PXM) assays are the T-cell and B-cell flow cytometry crossmatch (FCXM). The complement dependent cytotoxicity (CDC) crossmatch, with or without anti-human globulin (AHG), is still utilized by some laboratories due to its ability to detect strong complement binding antibodies, yet its use has decreased over the years (Putheti et al., 2022). Advancements in our ability to identify and characterize HLA antibodies using solid phase assays, in combination with changing deceased donor allocation schemes and improved HLA typing resolution, have shifted the paradigm from performing a PXM towards the use of a virtual crossmatch (VXM) as an organ offer screening tool and *in lieu* of a prospective PXM (Adler et al., 2021; Israni et al., 2021).

**TABLE 1:** Demographics of AC Survey participants in 2022.

Country	2022 AC-1	2022 AC-2
Argentina	2	2
Australia	4	4
Brazil	4	4
Canada	16	16
Chile	1	1
China	2	1
Colombia	2	2
Costa Rica	—	1
Guatemala	1	1
Hong Kong Special Administrative Region of China	1	1
Italy	1	1
Mexico	6	6
New Zealand	1	1
Panama	1	1
Peru	1	1
Poland	1	1
Puerto Rico	1	1
Qatar	1	1
Saudi Arabia	1	1
Singapore	1	1
Thailand	1	1
United States	106	107
Total	155	156

The VXM is not a physical laboratory test, but rather a compatibility assessment that relies on an interpretation of pre-transplant HLA laboratory test data. As such, the VXM assessment is not an “analyte” as opposed to a T-and B-cell crossmatch or Class I and Class II HLA antibody test. The VXM has been defined previously in (Morris et al., 2019) as an assessment of HLA compatibility between a donor and recipient based on the recipient’s anti-HLA antibody profile and the donor’s histocompatibility antigens. This definition will be applied henceforth. In the context of the PT survey, the VXM assessment was compared to the results of the physical crossmatch. There are several advantages to performing a VXM assessment *in lieu* of a prospective PXM. The efficiency of the allocation process is greatly improved due to a decrease in organ cold ischemia time, allowing for matching over a larger geographic area, as well as better access to transplant for highly sensitized patients (Bingaman et al., 2008; Johnson et al., 2016; Rohan et al., 2020; Puttarajappa et al., 2021). The VXM assessment is rapid, sensitive and does not require donor cell incubation with a recipient serum. Another important advantage is the absence of actual bench work, reduced on-call time, and reagent use, which decreases the overall operational cost of the HLA laboratory. Despite these many advantages, the VXM assessment has some limitations, which may restrict its utilization as a final compatibility assessment (i.e., without a prospective or retrospective PXM) in some cases. These limitations include the availability of a current serum, as defined by agreement between the laboratory and the transplant program, the presence of allele-specific antibodies in the absence of donor high-resolution HLA typing, donor HLA alleles that are not covered by the single antigen bead (SAB) panels, and limitations of the SAB assays themselves. For example, antibodies to shared eplets, multiple low level donor-specific antibodies (DSA) that may have an additive effect, inhibitory factors, false positive reactions due to denatured antigens, etc. In such cases, the VXM assessment may not provide accurate results and a PXM should be performed (Jani et al., 2017; Guidicelli et al., 2018; Greenshields and Liwski, 2019; Garcia-Sanchez et al., 2020; Sullivan et al., 2020; Kumar et al., 2021).

HLA laboratories may assess or predict compatibility differently based on their experience and practices. Laboratories differ in MFI cut-offs, approaches for analysis of antibody patterns and reactivity, physical crossmatch methodologies, servicing different transplant programs with different organ types, immunosuppression regimens and so on. This makes this compatibility assessment patient- and center-specific (Puttarajappa et al., 2023). Notably, the accuracy of the VXM assessment relies heavily on the quality of antibody test results. Given that the VXM assessment is used for clinical decision making, HLA laboratories must follow best practice guidelines to appropriately evaluate the immunologic risk to the patient (Tambur et al., 2018). Therefore, it was desirable to create an educational challenge that would help our global HLA community to compare detection of DSA, to understand how HLA laboratories are interpreting donor HLA typing and anti-HLA antibody profiles to predict a physical crossmatch result and assess transplant compatibility. These elements were missing from the ASHI PT surveys that only grade based on results of the physical crossmatch and antibody tests.

In this manuscript, we describe the ASHI PT Educational VXM Challenge as a model for creating educational content using PT survey data. We discuss the different components of the VXM challenge and highlight major findings and learning points acquired from the ASHI VXM challenges.

## Materials and methods

### Designing a VXM challenge

**FIGURE 1:**
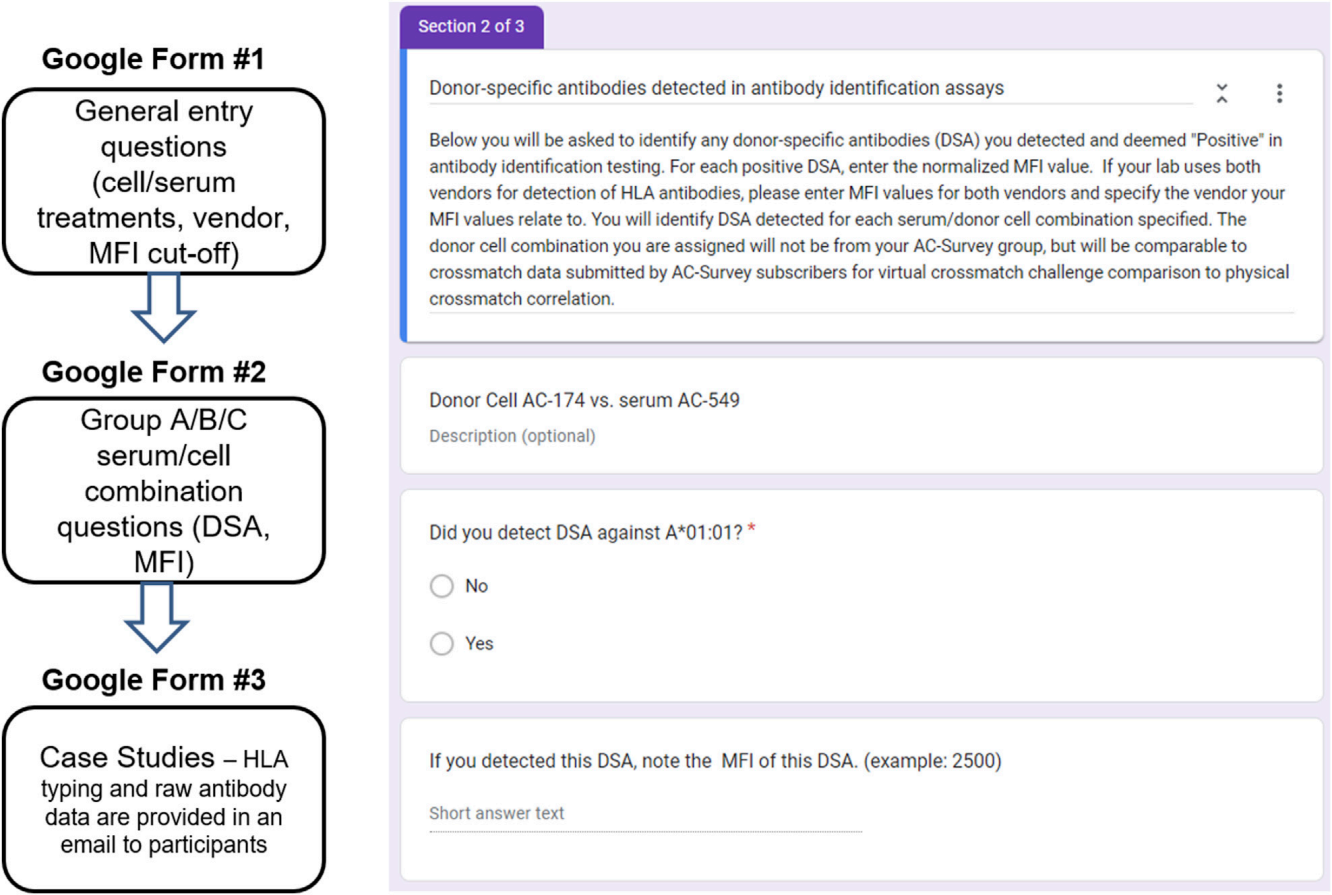
All VXM data were collected in Google Forms, exported, and analyzed in Microsoft Excel and/or GraphPad Prism. This challenge used PT data from the AC survey for educational purposes and was designed to have several components: General Entry questions, Cell/Serum Combination questions, and Case Studies. Right: An example for a Cell/Serum Combination Google Form.

**FIGURE 2:**
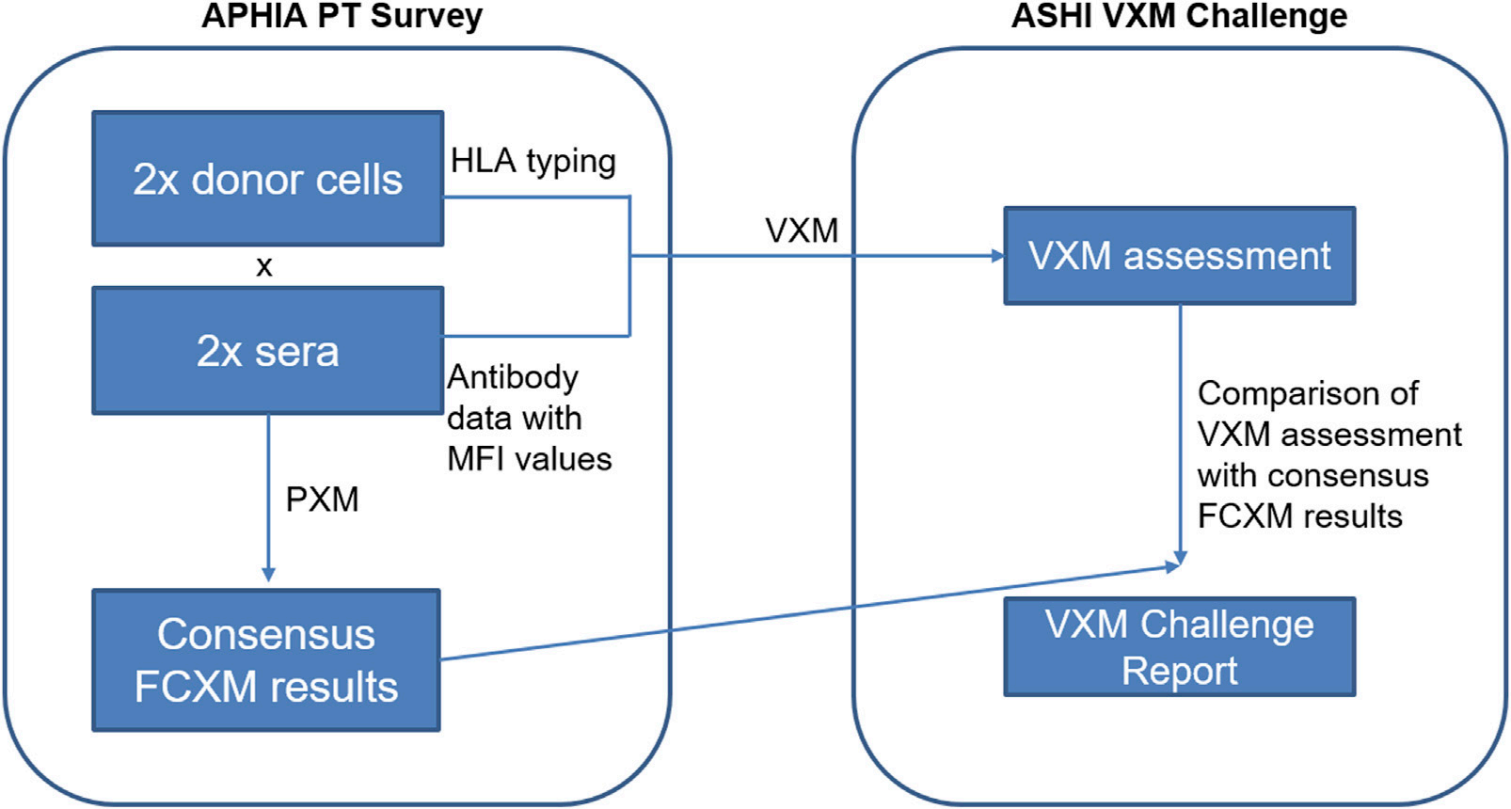
The International Case Study data consisted of two donor cells, each virtually crossmatched with two sera tested in the APHIA PT survey. Participants were provided with raw MFI data for the two serum samples and donor next-generation sequencing (NGS) typing for the two cell samples, and were asked to predict results of the FCXM for T- and B-cells based on the detected DSA for each serum/donor cell combination. Consensus PXM results were provided by APHIA retrospectively.

The PT Committee oversees the ASHI PT program with the goal of collaborating with HLA laboratories around the world to achieve the highest standards and continuous quality improvement in clinical testing and patient care. The committee strives to identify new PT challenge opportunities and to provide customized and comprehensive educational content. Given the increased use of the VXM assessment throughout our HLA community, the ASHI PT Executive Committee (EC) identified the need for a VXM Educational Challenge and launched the first challenge in 2018. This challenge was conducted in conjunction with the ASHI AC survey that is offered twice per year. The survey was built based on the grouping assignment for participants on the ASHI AC survey (Groups, A, B and C), where laboratories from each group received a different set of cells for crossmatch testing. The VXM Challenge participants were given a low/intermediate resolution HLA-A, B, Bw, C, DRB1, DRB345, DQA1, DQB1, DPA1 and DPB1 donor typing. Participants were required to use their own anti-HLA antibody data from the AC survey to perform a VXM assessment without knowledge of the PXM result. Serum/cell combinations for inclusion in the challenge were selected by the ASHI PT EC based on data from the AC survey. When possible, some of these combinations included challenging samples such as weak DSA, non-consensus physical crossmatch results, etc. Participant responses were collected in Google Forms after AC survey results were submitted but before AC Summary Reports with PXM results were published. The responses were exported and analyzed in Microsoft Excel and/or GraphPad Prism and comprehensive summary reports of analyzed data were written and reviewed by members of the ASHI PT EC. The challenge included three components (Figure 1), that contained questions, each allowing submission of information on different aspects of VXM assessment and/or laboratory practices:

1) General Entry questions: These questions were selected for the purpose of probing participants’ use of SAB panels for HLA antibody detection and identification, serum treatment for solid-phase assays, cell treatment with pronase for FCXM and mean fluorescence intensity (MFI) cut-off values for positive DSA. This section provided valuable information regarding common practices and trends across HLA laboratories.
2) Cell/Serum Combination questions: Participants were asked to identify DSA using data from the ASHI AC survey and to predict the outcome of a physical FCXM or complement dependent cytotoxic crossmatch (CDCXM). An example for participant groups and cell assignment is found in Table 2. HLA typing for donor cells is performed using real-time PCR single antigen bead resolution (RT-PCR SABR) trays and the results are provided to all participants of the AC survey, while the antibody data comes from each participating laboratory. MFI data for detected DSA is collected and analyzed to determine the variability in MFI values between different laboratories. VXM assessments from the three groups are compared to the physical CDCXM and FCXM results obtained from the AC survey. This section of the VXM Challenge also includes questions related to risk stratification (low, moderate or high risk), as well as offer acceptance criteria and provides information on whether participants would accept an organ offer based on their VXM assessment for different organ types. In later versions, participants were asked if they would recommend a PXM based on their VXM assessment.
3) Case Studies: Case studies may include real patient scenarios provided by an ASHI accredited HLA laboratory or include cases which are a result of collaboration between ASHI and a sister society, (e.g., Asia Pacific Histocompatibility and Immunogenetics Association - APHIA). For each case study, participants receive donor and recipient HLA typing data as well as recipient’s antibody identification data, and are asked to predict the results of the PXM. An example of a case study is illustrated in Figure 2 and was a result of a collaboration between ASHI and APHIA. The selected case studies emphasized the importance of evaluating current and historical antibody data, antibodies with low signal yet spread over multiple HLA specificities, donor specific anti-HLA antibodies with borderline strength, as well as detection of inhibitory factors in sera, to mimic the level of complexity that HLA laboratories face in real clinical situations. The goal of these case studies is to emphasize key VXM learning points from real cases and highlight them in the summary reports for participants.

**TABLE 2:** Number of 2022 VXM-1 Participants and cell assignments.

# Of responses per group	Cells used in AC-1 survey	Cells provided for VXM-1 challenge
Group A (N = 13)	AC-165, AC-166	AC-167 and AC-168 vs. AC-540
Group B (N = 10)	AC-167, AC-168	AC-165 and AC-170 vs. AC-540
Group C (N = 15)	AC-169, AC-170	AC-167 and AC-168 vs. AC-540
All Groups (N = 38)	n/a	APHIA C-05 and C-06 vs. Ser-07 and Ser-08

The 2022 VXM-1 challenge consisted of four AC-1 donor cells (AC-165, AC-167, AC-A68, AC-170), each virtually crossmatched with one serum (AC-540) tested in the 2022 AC-1 survey. Each group was assigned a donor cell that was not tested by this group for their cell-based crossmatch in the 2022 AC-1 survey.

### Correlation between VXM assessment and PXM

The ASHI VXM Challenge remains educational and therefore participants do not receive a grade for this effort. However, the results from the VXM assessments are compared to PXM results collected from the AC survey which is graded. In addition, reported DSA are compared to the consensus antibody results from the AC survey. Grading for the AC survey was performed as follows: The physical T-cell and B-cell FCXM and CDCXM were graded based on 80% consensus among participating laboratories. HLA class I and class II antibody specificities were graded separately. An antibody specificity reported by ≥ 90% of participants, either positive or negative, reached consensus. A minimum of 10 laboratories were required for grading. When less than 10 laboratories participated and when consensus requirements were not met the, results were not graded.

## Results

**FIGURE 3:**
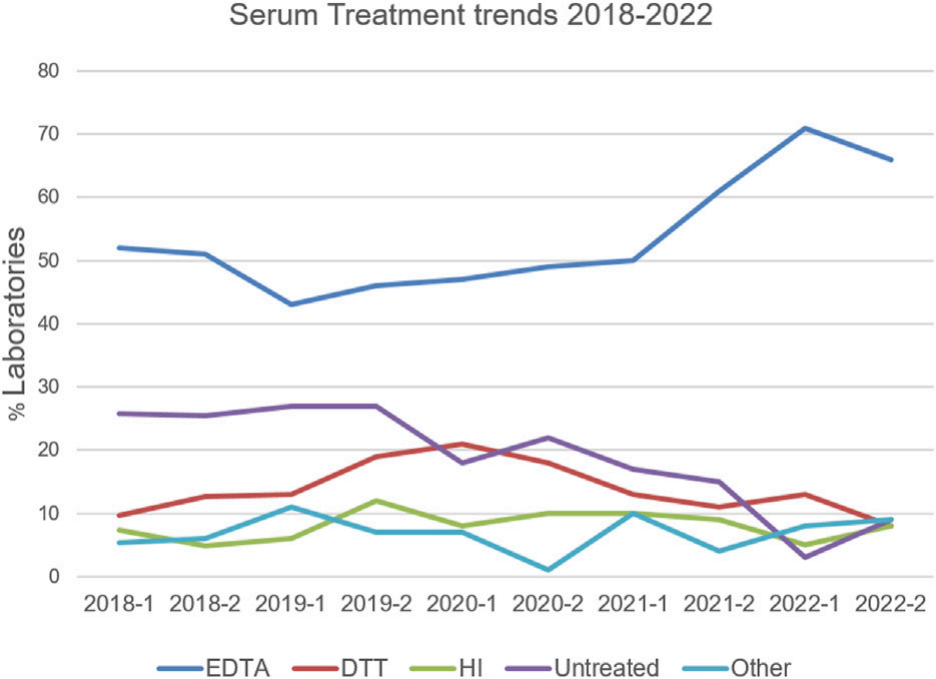
Serum treatment trends reported in the VXM Challenges between 2018 and 2022. Percentage of HLA laboratories using EDTA (blue), DTT (red), Heat Inactivation (HI; green), Untreated (purple) or Other (light blue) is depicted. “Other” included a combination of two treatments, dilutions, AdsorbOut beads, and Fetal Calf Serum treatment.

**FIGURE 4:**
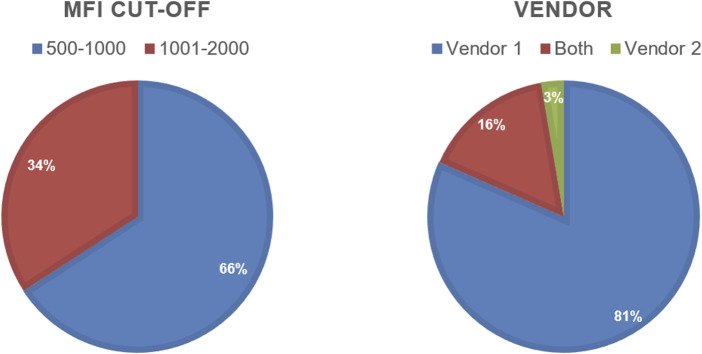
Pie charts showing MFI cut-off and Vendor use reported by the 2022 VXM-1 Challenge participants.

Over a 5-year period, 2975 virtual crossmatch assessments were reported. The median number of labs that participated in this non-graded challenge was 54 laboratories per challenge. Over time, as challenges have become more focused on “interesting” or educational cell/serum combinations, the number of VXM performed and reported per challenge by each laboratory decreased, and the participation rate was around 35% of the AC survey membership (Table 3). Serum treatment protocols were monitored overtime using the General Entry Question section of the VXM Challenge. Figure 3 depicts those trends and shows that the percentage of HLA laboratories that are not treating their sera has decreased over time (26% in 2018 versus 9% in 2022), while the use of Ethylenediaminetetraacetic acid (EDTA) as serum treatment has become more popular (52% of laboratories have reported the use of EDTA in 2018 versus 71% in 2022). The use of other treatments such as Heat Inactivation (HI) and Dithiothreitol (DTT) has fluctuated over time, although did not exhibit a specific trend. Most laboratories (>80%) reported the routine use of one SAB panel, which is distributed more heavily towards one particular vendor. Up to 16% reported the use of SAB panels from two vendors (Figure 4). Most laboratories reported using a cut-off for antibody detection. In 2022, for example, 66% of participating laboratories reported using cut-offs of 500–1,000 MFI to define positive specificities, while 34% reported using cut-offs of 1001–2000 MFI (Figure 4). Several laboratories reported locus specific cut-offs indicating higher cut-offs for HLA-C, HLA-DQ and HLA-DP loci, and some laboratories reported using different cut-offs based on organ type and urgency. Laboratories that did not use a hard cut-off mentioned that their analysis was based on antibody patterns and eplets.

**TABLE 3:** Characteristics of the VXM challenges from 2018 to 2022.

	2018		2019		2020		2021		2022	
Challenge	VXM-1	VXM-2	VXM-1	VXM-2	VXM-1	VXM-2	VXM-1	VXM-2	VXM-1	VXM-2
Laboratories participating, n (% of ASHI AC Survey laboratories)	84 (56%)	63 (41%)	65 (43%)	49 (32%)	54 (37%)	51 (43%)	58 (38%)	44 (30%)	38 (25%)	53^a^ (34%)
VXM cell/serum combinations assessed by each participant^b^	each lab had 5 sera/2 cells (840 VXMs)	each lab had 5 sera/1 cell (315 VXMs)	each lab had 5 sera/1 cell (325 VXMs)	each lab had 5 sera/1 cell (245 VXMs)	each lab had 5 sera/1 cell (270 VXMs)	each lab had 5 sera/1 cell (255 VXMs)	each lab had 3 sera/1 cell (174 VXMs)	each lab had 3 sera/1 cell (132 VXMs)	each lab had 1 serum/2 cells (76 VXMs)	each lab had 1 serum/1 cell (50 VXMs)
Case Studies	none	none	none	none	none	none	none	none	2 sera/2 cells (152 VXMs)	Case 1. 1 serum/1 cell
										Case 2. 2 sera/1 cell (141 VXMs)

^a^ 53 laboratories responded to the Data Entry Questions section of the VXM challenge. Of those 50 laboratories completed the cell/serum combination section and 47 laboratories completed the Case Study section of the VXM challenge.

^b^ The number of total VXM does not include CDCXM prediction due to the variability in CDC XM results availability.

### Detection of DSA and prediction of PXM

The participants of the VXM Challenge were asked to identify any DSA detected for each serum/donor cell combination with MFI values deemed “Positive” based on their established MFI cutoff. The percentage of laboratories reporting a specific DSA, mean MFI, Standard deviation (SD) and Coefficient of variation (CV) for cell/serum combinations from the 2021 ASHI VXM-1 Challenge can be seen in Table 4. In general, there were 2 types of predictions: 1) In the presence of consensus positive DSA, typically with a high MFI, the prediction of a positive PXM is fairly straight forward (e.g., cell AC-153 VS. serum AC-532); 2) In the presence of low level DSA, typically <3000 MFI (single or combined), there was a lack of concordance between the VXM and PXM (e.g., cell AC-153 VS. serum AC-534, T-cell FCXM and cell AC-153 VS. serum AC-531). A detailed explanation for specific discrepancies between VXM and PXM results is provided in the highlights section below.

**TABLE 4:** DSA reported for cell AC-153 with sera AC-531, 532, and 534.

Pair	DSA	%Labs reporting this DSA in the VXM Challenge	Mean MFI	SD	%CV	Consensus on AC survey	%Lab predicting a pos T-cell VXM	%Lab predicting a pos B-cell VXM	%Labs reporting a pos T-cell FCXM	%Labs reporting a pos B-cell FCXM
AC-153 VS. AC-531	B*14:02	36	918	218	24	No (35%)	32	28	81	79
	B*18:01	75	1820	524	29	No (71%)				
AC-153 VS. AC-532	DRB1*12:01	100	2560	735	29	Yes DR12 (99%)	0	100	3	100
	DRB1*13:04	93	4577	1881	41	Yes DR13 (100%)				
	DRB3*01:01	46	1135	697	61	Yes DR52 (94%)				
	DRB3*02:02	82	1390	562	40	Yes DR52 (94%)				
	DQA1*01:04/DQB1*05:01	82	10939	2104	19	Yes DQ5 (99%)				
AC-153 VS. AC-534	C*02:02	75	2122	934	44	No (68%)	17	86	7	100
	DQA1*01:04/DQB1*05:01	86	15164	6603	44	Yes DQ5 (100%)				

### Highlights from ASHI VXM challenges

Each VXM Challenge was unique and provided an opportunity to look at factors contributing to the prediction of a PXM. More recent challenges incorporated fewer AC Survey cell/serum combinations but included additional educational case studies. A few highlights from past ASHI VXM challenges are provided below.

#### Example 1: Low level DSA

**FIGURE 5:**
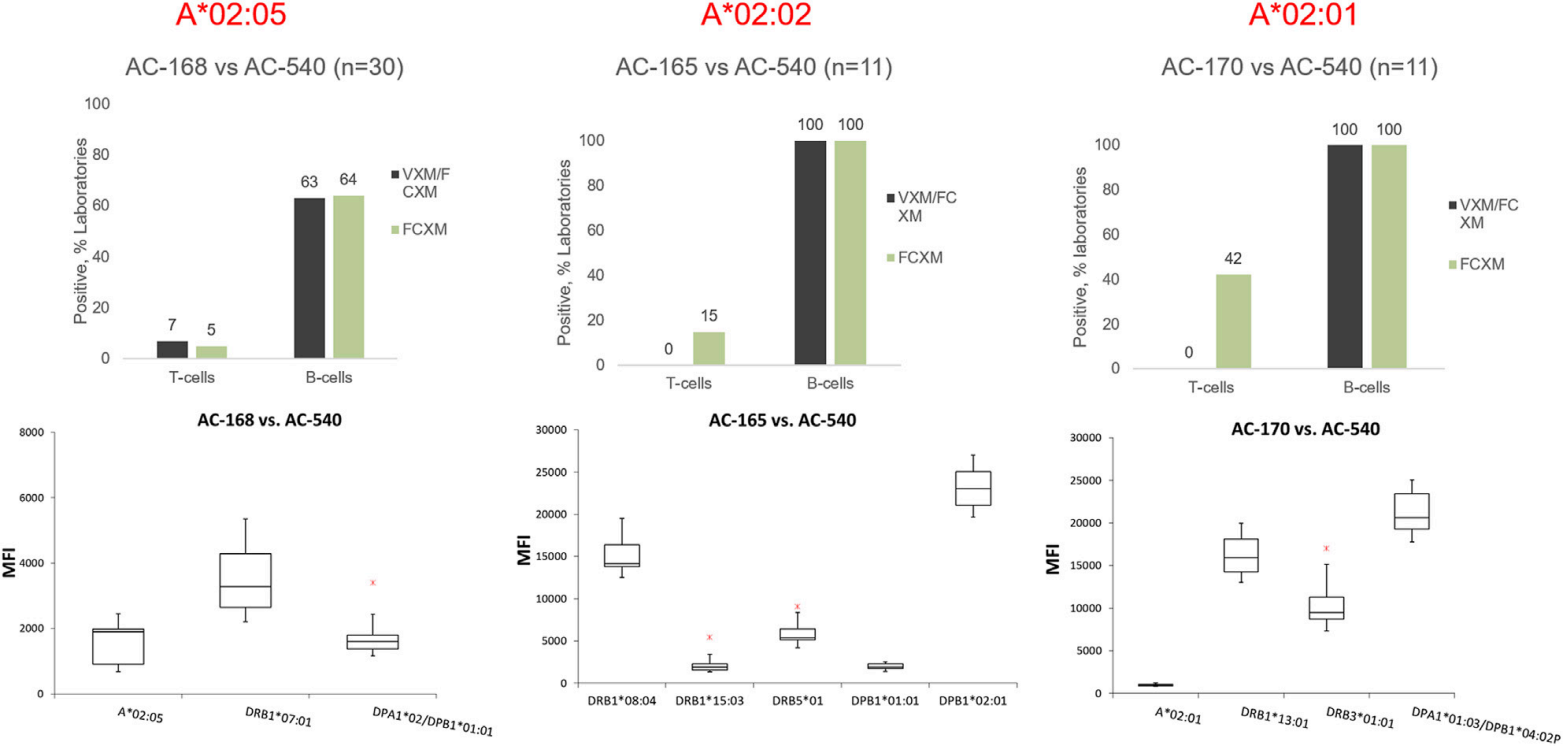
Top panel: Predicted (VXM/FCXM) and physical (FCXM) results for cells AC-168 (right), AC-165 (middle), and AC-170 (left) against serum AC-540. Cell AC-168 was typed as HLA-A*02:05, AC-165 was typed as HLA-A*02:02 and AC-170 was typed as HLA-A*02:01. Bottom panel: boxplots displaying the distribution of normalized MFI values reported by VXM Challenge participants against each cell based on a five number summary (“minimum” whisker, first quartile, median, third quartile, and “maximum” whisker). The * indicates the outliers. DSA reported by a single laboratory were not plotted. Only One lab reported an MFI value for A*02:02 (AC-165 vs. AC-540) and therefore, A*02:02 was not included in the graph.

The 2022 VXM-1 challenge included three donors typed as HLA-A*02, each donor carrying a different HLA-A*02 allele: AC-168 A*02:05, AC-165 A*02:02 and AC-170 A*02:01. The three donors were crossmatched against the serum AC-540. Participants were asked if they identified antibodies in serum AC-540, directed against each of the three HLA-A*02 donor alleles (A*02:05, A*02:02 and A*02:01). A total of 52 responses for all 3 donors were collected and of those, 42 laboratories (81%) indicated that they did not detect DSA against the corresponding A*02 donor allele in serum AC-540, some due to the donor allele not being present on the SAB panel, whereas 10 responses (19%) indicated that this serum was positive for DSA against HLA-A*02 with a mean MFI = 1377, SD = 600 and CV = 44. The MFI range using one vendor was 682–2441 and two laboratories also reported lower MFI values using a second vendor. Laboratories that reported MFI values commented that they used the HLA-A*02 beads present on their SAB panel to estimate the presence of antibody against donor A*02 alleles that were not represented on their single antigen bead panels (i.e., A*02:02 and A*02:05). A few laboratories also commented on stacking of the A*02 beads around the cut-off, or mentioned reactivity against the A2 CREG beads. Based on the low MFI value for the DSA, the majority of participants predicted that the T-cell FCXM would be negative with all three donors. The physical T-cell FCXM was consensus negative only for cells AC-165 (A*02:02) and AC-168 (A*02:05), while 42% of AC-1 survey participants reported a positive T-cell FCXM for cell AC-170 (A*02:01) (Figure 5). Of note, HLA-A2 antibody specificity was reported by 34% of AC survey participants and did not reach consensus. This example illustrates several points: first, VXM prediction lacks correlation with PXM in the presence of low level DSA. Second, when donor alleles are not represented on the SAB panel most laboratories use beads within the same antigenic group as surrogates. Since SAB panels from different vendors have different HLA allele coverage, it is recommended to tailor the panel to the donor alleles in order to accurately assess the presence of DSA. In ethnically diverse donor pools, utilizing an extended SAB panel that includes alleles commonly found in specific ethnic groups may be warranted. Another option is to test more than one panel/platform. In this example, a phenotype panel has representation of all three donor HLA-A*02 alleles and could be used to confirm reactivity.

#### Example 2: Impact of HLA-C antibodies

**FIGURE 6:**
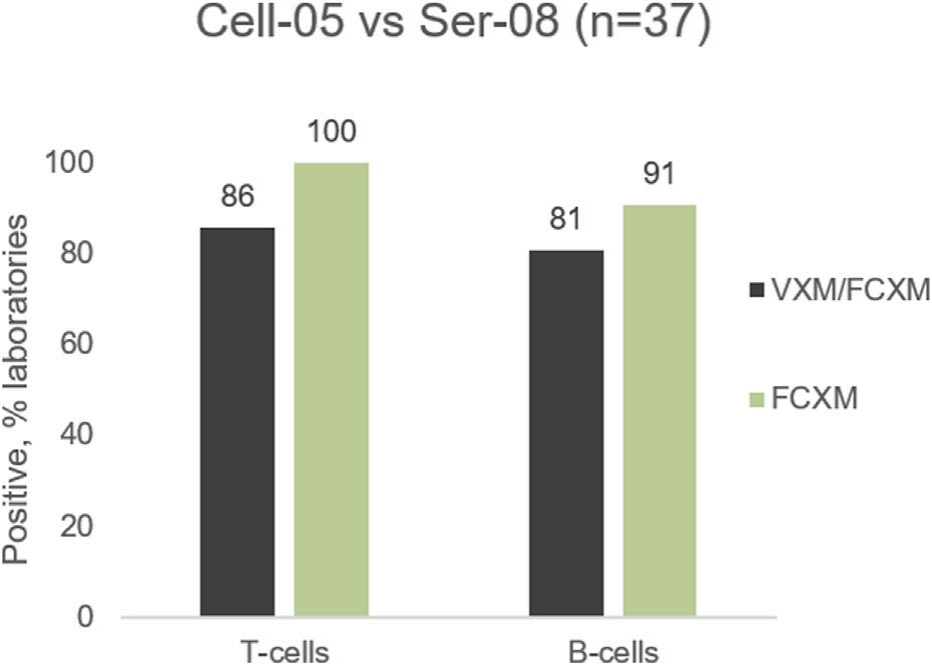
Predicted (VXM/FCXM) and physical (FCXM) results for Cell-05 against serum Ser-08.

The 2022 VXM-1 International Case Study included a cell/serum combination with DSA to HLA-Cw5 (MFI = 10,024), and Cw6 (MFI = 8924). Most laboratories accurately predicted a positive T- and B-cell FCXM. However, several laboratories seemed to underestimate the ability of anti-HLA-C antibodies to cause a positive FCXM, with 86% (32/37) of laboratories predicting a positive T-cell FCXM versus 100% (11/11) of laboratories reporting a positive physical T-cell FCXM and 81% (30/37) of laboratories predicting a positive B-cell FCXM versus 91% (10/11) of laboratories reporting a positive physical B-cell FCXM (Figure 6). Several laboratories commented that the prediction in this case is challenging due to the presence of C-locus antibodies only, as HLA-C has lower expression on the cell surface than HLA-A and HLA-B (Apps et al., 2015). As previously noted, data from the VXM Challenge indicates that laboratories tend to have higher cut-offs for calling C-locus antibodies. Despite the lower expression and the association of some C-locus antibodies with “non-specific” antibody patterns, this case highlights that these antibodies can be clinically relevant and therefore, a prospective PXM may be warranted to assess their ability to cause a positive crossmatch. On the other hand, Table 4 shows an example of lack of correlation between the VXM and T-cell FCXM in the presence of a DSA to HLA-C (cell AC-153 VS. serum AC-534). 75% of the VXM Challenge participants detected DSA against C*02:02 with an average MFI of 2122 in serum AC-534, and 83% of the participants correctly predicted a negative PXM in the presence of this low-level DSA, in correlation with the AC-1 survey negative consensus for the FCXM with AC-153 cells and AC-534 serum. However, 17% predicted a positive T-cell FCXM.

#### Example 3: Prediction of CDCXM

**FIGURE 7:**
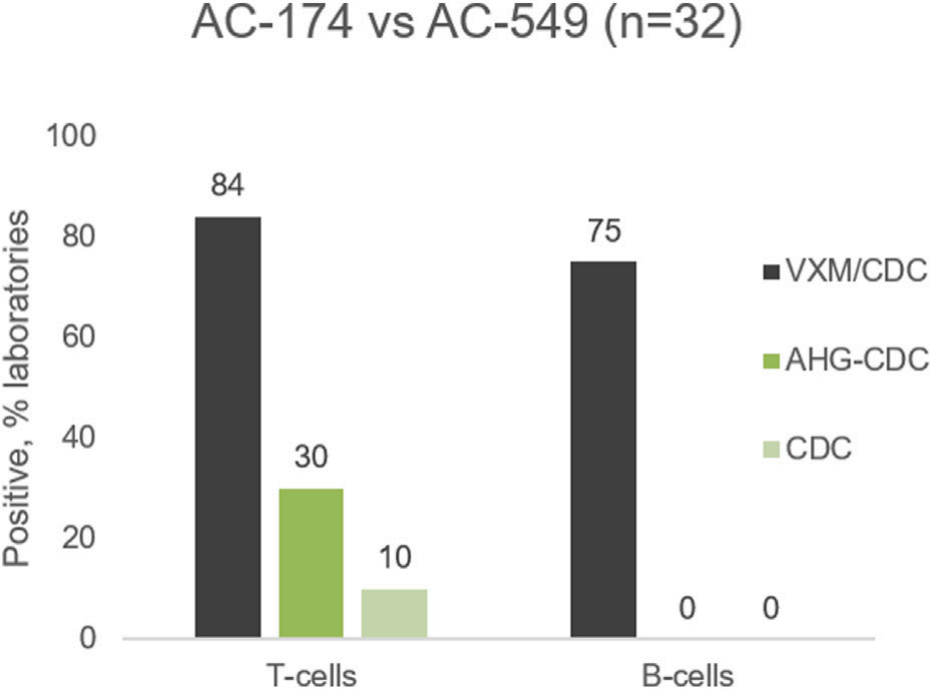
Predicted (VXM/CDC) and physical (AHG-CDC, CDC) results for cell AC-174 against serum AC-549.

The 2022 VXM-2 included a serum/cell combination, serum AC-549 against cell AC-174, with a consensus DSA to HLA-B8. A high mean MFI value of 12,235 was reported by the participants of the VXM challenge and may explain the positive prediction of T-cell and B-cell FCXM which agreed with the physical FCXM results obtained in the AC-2 survey. However, 84% and 75% of the VXM Challenge participants predicted positive T-cell and B-cell CDCXM, respectively, while consensus physical CDCXM was negative for both T-cells and B-cells (Figure 7). Only 10% of laboratories reported a positive T-cell CDCXM and only 30% of participants reported a positive T-cell AHG-CDCXM, even though 85% of the 2022 AC-2 Survey participants who utilized the C1q assay reported anti-HLA-B8 as complement fixing. This indicates that although C1q binding is often considered to be associated with antibody strength and a positive CDCXM (Zeevi et al., 2013; Tambur et al., 2015), predicting CDCXM results remains a challenge. Of note, the participants of the VXM Challenge did not have the C1q information at the time of VXM assessment, unless their laboratory performed the C1q assay. This information was available to participants only after the Challenge has been concluded. The high percentage of participants predicting a positive CDCXM in this case suggests that CDCXM results should not be predicted based on MFI values alone.

#### Example 4: Antibody against a shared eplet

**FIGURE 8:**
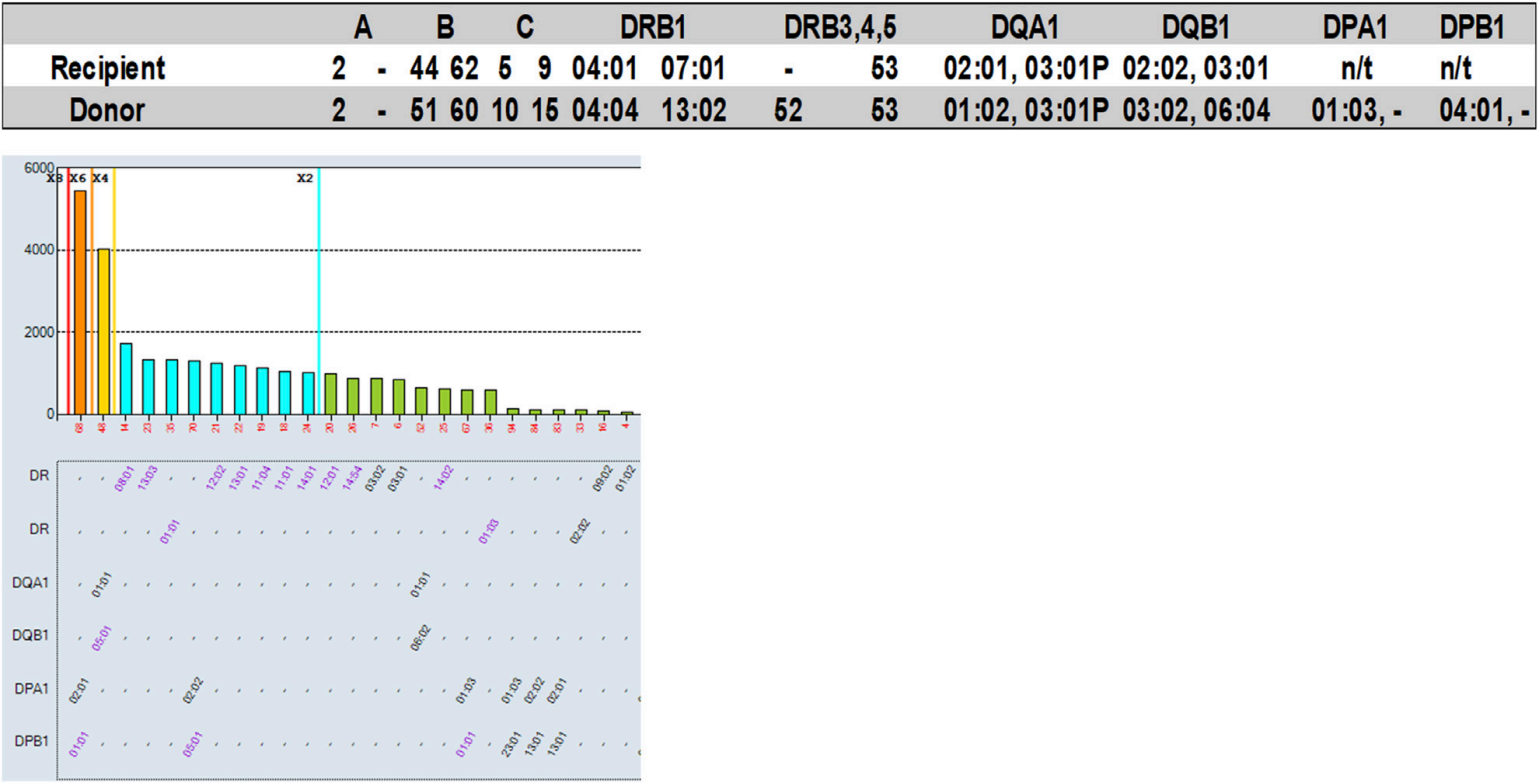
Participants of the 2022 VXM-2 Challenge were provided with recipient’s raw MFI antibody data (one serum sample) and donor HLA typing, and were asked to predict results of a FCXM for T- and B-cells based on detected DSA for the serum/donor cell combination. A total of 47 participants responded to this section of the challenge. Top: Recipient and donor HLA typing results. Bottom: HLA Class II SAB results.

One of the 2022 VXM-2 Case Studies included a donor typed as DRB1*13:02, an allele which is not present on the single antigen bead panel that was provided to the participants with this case study. Other DRB1*13 alleles had low normalized MFI values (DRB1*13:01 = 1,201; DRB1*13:03 = 1,336). 98% of participants correctly predicted a negative T-cell FCXM in the absence of HLA class I DSA. 26 out of 47 participants (55%) predicted a negative B-cell FCXM, 8 (17%) predicted a positive B-cell FCXM and 13 (28%) responded “Other” and commented that further testing would be required to determine compatibility in this case, either by running this sample on a different panel that covers the DRB1*13:02 allele, or by running a PXM. Several laboratories responded that the B-cell FCXM is likely to be negative assuming that the reactivity of the antibody to DRB1*13:02 is similar to the reactivity of the other DRB1*13 antibodies. Other laboratories noted that an antibody against a shared eplet might be present based on a reactivity pattern that included all the DR52 associated DRB1 beads stacking with low level MFI (Figure 8), and therefore the B-cell FCXM might be positive, and a few laboratories commented that the B-cell FCXM prediction is indeterminate. Based on their prediction most laboratories (64%) responded that a physical FCXM would be requested in this scenario. 17% of the participants responded that they would not request a physical FCXM and 19% responded “Other” indicating that a crossmatch would or would not be requested depending on the specific scenario. Interestingly, the physical FCXM for this case study was B-cell positive, despite the low MFI value of the surrogate DRB1*13 single antigen beads. As mentioned by several participants this B-cell FCXM positivity is likely due to the presence of an antibody against a shared eplet, although this phenomenon has been questioned by other groups and there may be additional or other factors contributing to the lack of correlation between SAB assays and FCXM results (Claisse et al., 2022). This reactivity could be explained by eplet 96HK which is present on DR8,11,12,13,14,17,18 but not on DR52 or by a combination of eplet 11STS which is present on DR11,13,14,17,18 but not the DR52 and eplet 16Y, which is present on DR8 and specific alleles of DR12 and DR14. Another example of this phenomenon can be seen in Table 4. For serum AC-531 vs. cell AC-153, a DSA against B*14:02 (B65) was reported by 36% of the VXM Challenge participants with a mean MFI value of 918, and a DSA against B*18:01 (B18) was reported by 75% of the VXM Challenge participants with a mean MFI value of 1820. Based on these results, 32% of laboratories predicted a positive T-cell FCXM and 28% of participants predicted a positive B-cell FCXM. However, the consensus result for the physical FCXM was T-cell positive. B-cell FCXM results were not graded due to lack of consensus, but 79% of laboratories reported a positive B-cell FCXM. This discrepancy is most likely due to the presence of an antibody against the Bw6 motif, which is expressed by both B65 and B18. These examples indicate that MFI cannot be trusted as accurate in cases of a shared eplet, and that weak shared eplets should trigger a PXM.

#### Example 5: Assessment of risk

**FIGURE 9:**
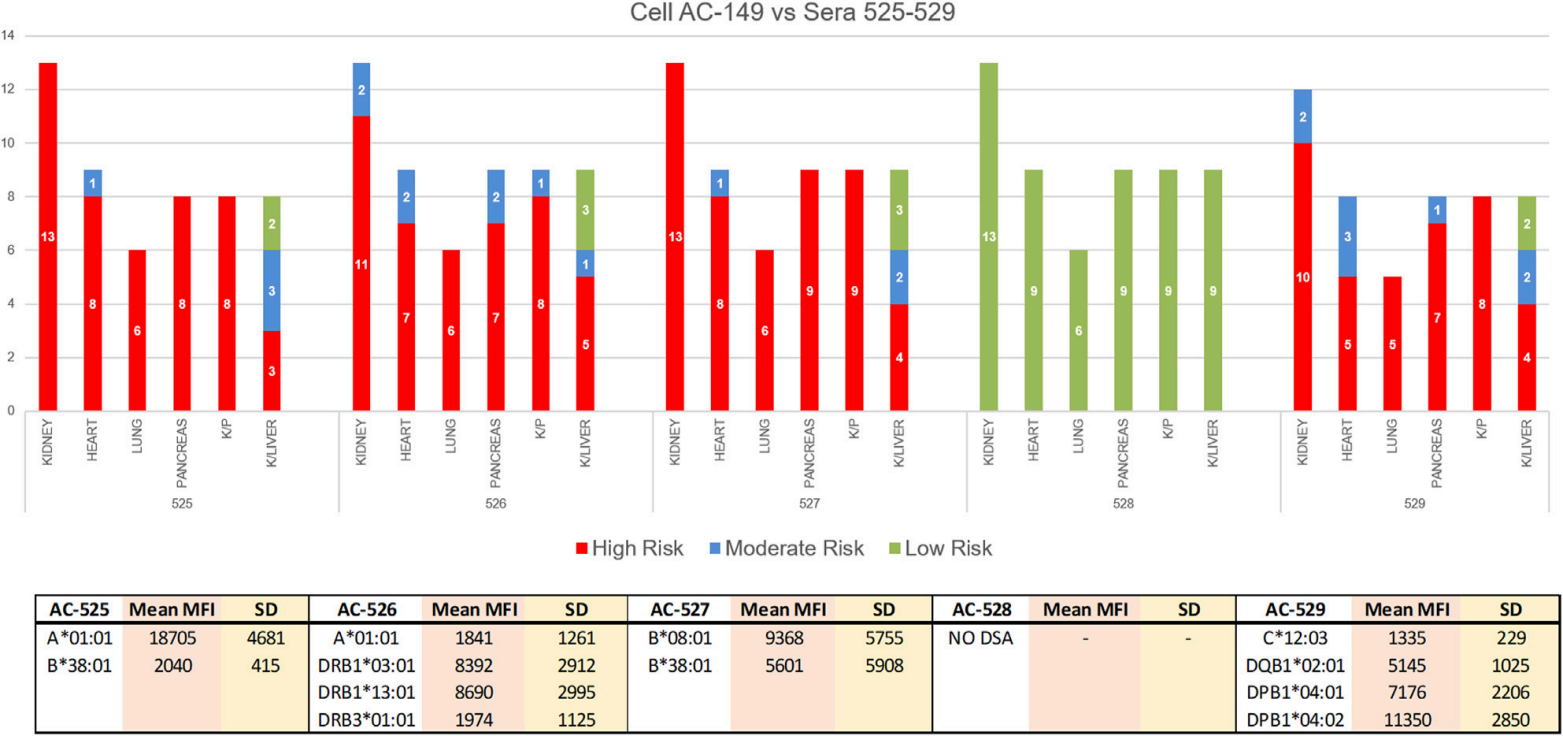
In the 2020 VXM-2 challenge, participating laboratories were asked to use the data from the VXM to provide a risk assessment in the case of kidney, heart, lung, pancreas, kidney/pancreas and kidney/liver transplantation. The responses are depicted in a bar graph; Red = High Risk, Blue = Moderate Risk, Green = Low Risk. For all sera/cell combinations, variability in risk assessment was mostly observed for kidney/liver transplantation. The bottom table is showing mean MFI values and standard deviation (SD) for DSA against cell AC-149 in sera AC-525-529.

In the 2020 VXM-2 challenge, participating laboratories were asked to use the data from the VXM to provide a risk assessment in the case of a kidney, heart, lung, pancreas, kidney/pancreas and kidney/liver transplant. For all sera/cell combinations, variability in risk assessment was mostly observed for kidney/liver transplantation (Figure 9). Specifically, serum AC-525 demonstrated DSA against cell AC-149: anti-HLA-A1 with mean MFI = 18,705, and anti-HLA-B38 with mean MFI = 2,040. All VXM Challenge participants assessed this donor/recipient pair as “High Risk” for kidney, heart, lung, pancreas and kidney/pancreas transplantation with the exception of one participant assessing this pair as “Moderate Risk” for kidney transplantation. In contrast, of the 8 participants assessing this pair for a kidney/liver transplantation, 3 scored this pair “High Risk,” 3 “Moderate Risk” and 2 “Low Risk,” indicating that VXM may vary based on center’s risk tolerance. Of note, the PXM for this serum/cell combination was reported T- and B-cell positive by 100% of laboratories using FCXM and T-cell positive by 55% laboratories using AHG-CDC, while, CDC T- and B-cell crossmatches were consensus negative.

## Discussion

The ASHI VXM Educational Challenge collects data from HLA laboratories across a broad geographical area on which assays laboratories are using for performing VXM assessment (i.e., SAB antibody panels), how they treat serum and cells, and what criteria they use for calling positive DSA. In addition, the challenge collects DSA MFI data and provides information on participants’ assessment of risk and criteria for accepting organ offers for renal versus non-renal transplant candidates. DSA MFI data reported by VXM Challenge participants demonstrated variability consistent with previously published data (Reed et al., 2013). These data could be used by laboratories to assess performance and determine whether any changes to their testing protocol are needed.

Despite the increased use of VXM in clinical practice, the participation in the VXM Educational Challenge has slightly decreased overtime, with a typical response rate of 30%–40% of laboratories enrolled in the ASHI AC Survey. This decrease could be due to the fact that the participation in this challenge is optional and not graded, or due to the length of the survey, which factored into the decision to reduce the number of cell/serum combinations per challenge. Each challenge was unique and varied in the number of cell/serum combinations and Case Studies given to participants and was designed to address different clinical scenarios encountered by HLA laboratories in their everyday practice. Our data shows that the percentage of HLA laboratories using serum treatments has increased from 2018 to 2022, with EDTA being the most used serum treatment. These data allow the PT EC to monitor changes in HLA laboratory practices over time. Overall, the data from VXM challenges demonstrate that in the presence of antibodies which are consensus-positive by SAB, most laboratories can predict a FCXM result more accurately than a CDCXM result. This could be due to CDCXM requiring additional crosslinking by multiple DSA of high titer for effective complement mediated cell death (Diebolder et al., 2014; Tambur et al., 2015). Virtual assessment guidelines may need to consider the antibody titers and correlation with C1q assay results to increase the accuracy of physical CDCXM result prediction. Although prediction of FCXM was generally more accurate, it became more difficult in the presence of low level DSA which often did not reach consensus on the ASHI AC Survey, when the DSA was aimed against donor antigens that are not present on the SAB panel (Kumar et al., 2021), and when an antibody against a shared eplet was suspected (Garcia-Sanchez et al., 2020). These limitations, for example, lack of antigen/allele representation on the single antigen bead assay, may result in unintentional exclusion from transplantation. In addition, the reverse may also occur (i.e., proceeding with a transplant in the presence of an undetected DSA). Tools such as the HLA eplet registry and HLA MatchMaker can be used to identify eplets present on alleles that are not included in SAB panels and common antigens that share these eplets can be used as surrogates when analyzing SAB reactivity patterns (eplet-based analysis). Alternatively, the use of expanded panels may help resolve some of these issues.

Some laboratories prefer to use a VXM assessment-only approach to select transplant candidates. Considerations for the exclusion of patients as a result of conservative evaluation of a VXM due to low or unclear reactivity on the SAB may disenfranchise a category of patients. Limitations of the SAB assay include false negative and false positive results and inability to accurately predict results of PXM in the presence of more than one weak DSA or DSA directed against shared eplets. Therefore, laboratories are encouraged to perform VXM assessment based on comprehensive evaluation of different test methods and analyses, including screening and phenotype assays and/or surrogate crossmatches, as well as eplet analysis. Laboratories should define which cases require a PXM in their transplant agreements, such as in highly sensitized patients.

Our VXM Assessment Challenge with follow-up PXM allows participants to determine, using their center practice guidelines, if their positive VXM assessment interpretation of the potential DSA would have led to a negative PXM. These VXM challenges are essential to educate our Histocompatibility community to increase awareness and understanding on how to fine-tune the correlation between the VXM assessment and PXM, which is critical for transplant equity.

FCXM sensitivity can be impacted by multiple factors including test protocols which are variable across laboratories (e.g., cell to serum ratio especially impacts samples showing weak DSA reactivity, cell treatment, etc.) (Jaramillo et al., 2018), cell source (blood vs. spleen vs. lymph nodes, as well as deceased vs. living donors) (Badders et al., 2015), and HLA expression level on the cell surface, which can be assessed either at the protein level or at the transcript level. New technologies for the assessment of HLA allele transcript levels, in combination with patients’ SAB data and donor HLA typing, could provide more granular data for VXM assessment and risk stratification (Cornaby et al., 2022). Importantly, participants of the ASHI VXM Challenge are instructed to respond based on their center practices. HLA laboratories utilize different DSA cut-offs, cell and serum treatments, and antibody SAB panels, all of which impact the prediction of a PXM. The VXM Challenge provides opportunity for participating laboratories to assess the analytic validity of their solid phase assay, improve their prediction capabilities, make VXM assessment more accurate and put policies in place for when a VXM can be used *in lieu* of a PXM.

The VXM Challenge has some limitations: 1) Transplant centers vary in the risk they are willing to take based on organ type, transplant volumes and patient clinical characteristics. The VXM Challenge does not collect data on transplant volumes and organ type, however, the risk stratification section includes separate questions for renal versus non-renal transplant candidates and some challenges included organ-specific risk assessment questions. 2) It is important to remember that VXM Challenge participants do not have clinical information such as history of sensitizing events (e.g., pregnancies, prior transplants, transfusions, etc.) and “patient” typing, with the exception of Case Studies, which may limit their ability to accurately predict PXM results. Therefore, for the purpose of this challenge, participants were instructed to assume that there is no matching between HLA typings of donor and recipient pairs, each recipient has had no significant clinical events to consider, the participants have tested current serum samples and the patient’s anti-HLA antibody testing history is consistent. In addition, while the participants of the VXM utilize their own raw antibody data to perform VXM assessment for the cell/serum combinations, the antibody data for the Case Studies is provided in excel spreadsheets, which may limit their ability to perform eplet analysis. In the future, comma-separated values (CSV) files may be used to address this issue. 3) The VXM Challenge does not collect raw data on all single antigen beads from participants, it only collects DSA bead data, and therefore the analysis of antibody and eplet patterns relies on comments provided the participants. The participants are encouraged to report pertinent observations such as eplet reactivity in the Google Form. 4) Response rate is about a third of all AC Survey participants. However, even with this response rate the challenge had a median of more than 50 participants per challenge, which translates into more than 100 VXMs per challenge.

In conclusion, the ASHI VXM Educational Challenge serves as a valuable tool that highlights the strengths and pitfalls of the VXM assessment and reveals differences in testing and results interpretation in participating HLA laboratories. This is particularly important since understanding the collective mindset of HLA laboratories during a challenging or borderline case can help all members of the HLA community when they are faced with a difficult assessment and are unable to perform a PXM. In an era where 26% of HLA laboratories use VXM followed by a retrospective PXM, and 8% rely solely on VXM to determine donor-recipient compatibility for deceased donor kidney transplants (Puttarajappa et al., 2023), it is imperative that we collect data that can shed light on when it is most advantageous and safe to utilize VXM and when a PXM is still preferable.

## Data Availability

The raw data supporting the conclusion of this article will be made available by the authors, without undue reservation.

## Abbreviations

AC, Antibody and crossmatching; AHG, Anti-human globulin; APHIA, Asia Pacific Histocompatibility and Immunogenetics Association; ASHI, American Society for Histocompatibility and Immunogenetics; CDC, Complement dependent cytotoxicity; CDCXM, Complement dependent cytotoxicity crossmatch; CSV, comma-separated values; DSA, Donor specific antibodies; DTT, Dithiothreitol; EC, Executive Committee; EDTA, Ethylenediaminetetraacetic acid; FCXM, Flow cytometry crossmatch; HI, Heat Inactivation; HLA, Human leukocyte antigen; MFI, Mean fluorescence intensity; NGS, Next-generation sequencing; PT, Proficiency testing; PXM, Physical crossmatch; RT-PCR SABR, Real-time PCR single antigen bead resolution; SAB, Single antigen bead; VXM, Virtual crossmatch.
